# Hypogonadism and its associated factors among adult male type 2 diabetes mellitus patients at the university of gondar comprehensive specialized hospital, Northwest Ethiopia 2024: A comparative cross-sectional study

**DOI:** 10.1371/journal.pone.0329784

**Published:** 2025-08-14

**Authors:** Arega Zenaw, Elias Chane, Getnet Fetene, Habtamu Wondifraw, Amare Mekuanint, Yonatan Kinde, Temesgen Kassie, Kassaw Demeke, Alebachew Fasil, Abebaw Worede

**Affiliations:** 1 Department of Clinical Chemistry, School of Biomedical and Laboratory Sciences, College of Medicine and Health Sciences, University of Gondar, Gondar, Ethiopia; 2 Department of clinical chemistry, School of Biomedical and Laboratory Science, college of medicine and health science, Debre Markos University, Debre Markos, Ethiopia; 3 Adelaide Medical School, University of Adelaide, Adelaide, South Australia, Australia; Federal University of Minas Gerais: Universidade Federal de Minas Gerais, BRAZIL

## Abstract

**Introduction:**

Hypogonadism is an established complication in male patients with type 2 diabetes mellitus, potentially exacerbating metabolic dysregulation and impairing quality of life. Global studies have documented an elevated prevalence of hypogonadism among diabetic individuals. However, there remains a scarcity of data regarding the prevalence and contributing factors of hypogonadism in patients with type 2 diabetes mellitus in Ethiopia, particularly in the Gondar region.

**Methods and Materials:**

This comparative cross-sectional study included 330 participants (165 with type 2 diabetes mellitus and 165 age-matched controls), selected via systematic random sampling from June 27 to August 20, 2024. Data were collected through structured questionnaires and 5 mL blood samples were obtained by laboratory technologist. Analysis using SPSS version 25 involved descriptive statistics, independent t-tests, and binary logistic regression to identify factors associated with hypogonadism, with significance set at p < 0.05.

**Result:**

The mean age of participants was 57 ± 8.9 years in the type 2 diabetes mellitus (T2DM) group and 55 ± 8.6 years among controls. Hypogonadism was identified in 30.3% of individuals with T2DM, compared to 7.2% in the control group. Multivariable logistic regression revealed that T2DM, elevated fasting blood glucose, advancing age, dys-regulated high-density lipoprotein levels, and obesity were independently associated with increased odds of hypogonadism.

**Conclusion and Recommendation:**

The prevalence of hypogonadism was markedly higher in individuals with type 2 diabetes mellitus compared to the control group, concomitant with a significantly lower mean testosterone level. Multivariate analyses identified fasting blood glucose, high-density lipoprotein levels, advanced age, the presence of diabetes, and obesity as independent risk factors for hypogonadism. Notably, modifiable factors such as obesity, in conjunction with key metabolic indicators, underscore potential targets for intervention to alleviate the burden of hypogonadism in diabetic patients.

## Introduction

Diabetes mellitus (DM) is a category of metabolic disorders caused by an impairment in the secretion, or action of insulin that leads to hyperglycemia [1]. It can be majorly classified into two types: type 1 DM(insulin dependent) and type 2 DM(insulin independent) [2]. T2DM can persist for a long time without being identified and frequently appear over a period of years, over time this condition leads to a serious damage to heart, blood vessel, eye, kidney, nerve, and testicles [3]. Hypogonadism is an endocrine disorder caused by the testes incapacity to generate testosterone, a primary male sex hormone regulating erectile function, libido and fertility [4]. Hypogonadism is significantly overlooked complication of T2DM.

The exact cause of hypogonadism in males with T2DM is not fully understood, but several contributing factors have been postulated. Firstly, insulin resistance, an important characteristic of T2DM, seems to be an identifiable factor. The action of insulin and responsiveness to insulin in the brain and testis are necessary for the adequate function of the hypothalamo-hypophyseal axis, so the hypothalamo-hypophyseal axis becomes impaired and causes low production of testosterone due to insulin resistance [5]. This is supported by the studies conducted on animal models, comparing male diabetic rats to non-diabetic controls, the frequency and amplitude of the LH pulse for diabetic rats were reduced by 50%, but, when insulin therapy is administered twice a day, the frequency of LH was fully corrected in diabetic rat [6].

Obesity can also be another factor to cause hypogonadism in males with T2DM. From adipose tissue of obese individuals there are an excess formation of aromatase enzyme, leptin hormone and pro-inflammatory cytokines [7]. There is a conversion of serum testosterone levels to estradiol due to the action of the aromatase enzyme through the process of aromatization. This conversion leads to lower blood testosterone levels and acts as negative feedback on the hypothalamic-pituitary-gonadal axis (HPG) and gonadotropin releasing hormone [7,8].

Despite, there is an excessive formation of the hormone leptin in the white adipose tissue. However, obesity impaired the effect of excess leptin in the neuron [7]. Leptin is essential for the normal pulsatile secretory function of GnRH neurons in the arcuate nucleus of the hypothalamus. The permissive effect of leptin on GnRH pulsatile is mediated through kisspeptin-expressing neurons in the arcuate nucleus of the hypothalamus [9]. Leptin receptors are present on kisspeptin neurons, indicating that leptin can directly influence the activity of these neurons. Leptin acts on kisspeptin neurons to regulate the release of kisspeptin. This can stimulate the release of GnRH, which in turn regulates the secretion of LH and FSH [10].

Pro-inflammatory cytokines play a role in the development of hypogonadism. Several studies have shown that adipo-cytokines, such as tumor necrosis factor-alpha and interleukin-1 beta, can suppress GnRH and LH production in vitro in experimental animals [11,12]. Numerous studies have revealed that among male T2DM patients, age, waist circumference (WC), BMI, hyperglycemia, smoking and dyslipidemia are significant factors that are strongly linked to hypogonadism [13,14].

There is a growing global concern about the high prevalence of hypogonadism, which has been reported to range from 12% to 78% capturing public health attention [15,16]. According to different studies, male hypogonadism and its concomitant conditions are linked to worse clinical results and a greater financial strain on healthcare systems [17,18]. The most known psychological and socio cultural impacts linked to male hypogonadism are anxiety, bad social interaction, breaking relationship with partner due to inadequate sexual intercourse, and depression [19,20].

The Androgen Deficiency in the Aging Male (ADAM) questionnaire, created at Saint Louis University, has been used extensively since 2000 as a screening tool to identify men who may be at risk for androgen deficiency. Its usefulness as a screening test was highlighted by its 88% sensitivity. However, at 60%, it was demonstrated to be less specific [21]. These findings suggest that the ADAM questionnaire has to be changed to better support the early detection of hypogonadism in patients.

Scholars suggested that early identification and provision of treatment decreases T2DM related hypogonadism. Additionally, identifying the contributing factors for hypogonadism helps to decrease the long term complications and improve patient’s quality of life. However, there is limited evidence of magnitude and contributing factors of hypogonadism. Hence, this study aimed to assess the prevalence and its associated factors of hypogonadism among T2DM patients in University of Gondar Comprehensive Specialized Hospital. Despite, this study was done in Ethiopia specifically AdisAbeba, that characterized by the more urbanization and sedentary life style as compared to our study area.

## Methods and materials

### Study area, study design and period

This study was conducted at the chronic patient care clinic of the University of Gondar Comprehensive Specialized Hospital (UoGCSH) in Gondar, Ethiopia, during 2024. UoGCSH is situated in the northwestern highlands of Ethiopia, within the Amhara Regional State, approximately 656 km from Addis Ababa, the nation’s capital. A comparative cross-sectional study design was employed, with data collection occurring from June 27 to August 20, 2024.

### Study populations

A total of 330 participants were enrolled and stratified into two groups. The first group comprised patients with T2DM, while the second group consisted of age-matched, ostensibly healthy controls. The control group was recruited from individuals visiting the UoGCSH chronic patient care clinic as well as from UoGCSH staff members. All participants met the inclusion criteria, and data were collected between June 27 and August 20, 2024.

### Eligibility criteria

#### Inclusion criteria.

Adult male patients with a confirmed diagnosis of T2DM within the preceding six months, attending the chronic patient care clinic at UoGCSH, and who provided voluntary informed consent were included in the study. Additionally, apparently healthy adult male individuals, identified as normoglycemic through screening tests and who similarly consented voluntarily to participate, were also enrolled.

#### Exclusion criteria.

Participants with a documented history of testicular surgery, chronic liver disease, or end-stage renal disease, as determined through medical record review, were excluded from the study. Additionally, individuals who tested positive for HIV/AIDS during the screening process were not included. Further exclusion criteria encompassed the use of specific medications known to influence hormonal balance, such as opioids, glucocorticoids, and testosterone replacement therapy (TRT). Moreover, individuals with mental health disorders or hearing impairments that could hinder their ability to provide reliable information were also excluded.

### Sample size and sampling technique

The sample size was determined by using a mean difference formula from previous study done in Sudan [16].

Systematic random sampling was employed to select study participants from the diabetic follow-up cohort at the University of Gondar Comprehensive Specialized Hospital (UoG CSH) chronic care clinic. Based on clinic records, approximately 12,000 patients were seen annually for diabetes management, with an estimated 2,000 expected during the two-month data collection period. The sampling interval (k) was determined by dividing the estimated population (N) by the required sample size (n), yielding. K = N/n. K = 2000/346 ≈ 5.8, rounded to 6. Accordingly, every 6^th^ patient presenting to the clinic was recruited. The initial participant was selected by a simple random lottery method from among the first six eligible patients to ensure randomness at the start of the sampling sequence.

### Data collection procedure

A standardized data collection tool, comprising a structured questionnaire and checklist, was developed for the study. To assess symptoms of testosterone deficiency, the internationally validated Androgen Deficiency in the Aging Male (ADAM) questionnaire was adapted [21]. The questionnaire was initially prepared in English and subsequently translated into Amharic, the local language. It consisted of three main sections addressing socio-demographic, clinical, and behavioral characteristics. A pretest was conducted on 5% of the sample at Maraki Health Center to evaluate the clarity, consistency, and reliability of the tool.

Eligible participants were identified based on predefined inclusion criteria and were provided with detailed information regarding the study objectives. Informed written consent was obtained from all individuals who agreed to participate. Prior to data collection, screening procedures were conducted: fasting blood glucose (FBS) testing was used to confirm eligibility among controls, while HIV testing was performed for both groups. Participants who met the eligibility criteria were then interviewed using the structured questionnaire. The adopted ADAM questionnaire have 10 questions that evaluates three main domains, namely energy, mood, and sexual function. If the study participant responded “yes” to questions 9 and 10 or to any other 3 questions, the study participant considered as ADAM positive; if not, ADAM negative.

Anthropometric measurements, including height and weight, were obtained using a stadiometer and bioelectrical impedance analyzer (Seca, Germany), respectively. Participants removed shoes, hats, and bulky clothing during measurement, and body mass index (BMI) was calculated as weight (kg) divided by height squared (m²), with classification into underweight, normal weight, and obese categories. Waist circumference (WC) was measured at the midpoint between the lower rib margin and iliac crest using a non-stretchable tape. Blood pressure was assessed using a mercury sphygmomanometer (India), with the average of two readings taken after a 10-minute rest.

Clinical data, including diabetes complications, treatment duration, and medication type, were extracted from medical records using a standardized checklist. Behavioral characteristics—such as physical activity, alcohol and coffee consumption, diet, stress, and smoking—were assessed via structured questionnaire. Physical activity was defined per WHO guidelines (≥150 minutes/week). Stress was measured using the Perceived Stress Scale. Alcohol use was categorized as current drinker, non-drinker, or ex-drinker; smoking status as current, ex-smoker (ceased >6 months), or never smoker. Following the interview, 5 mL of fasting venous blood was collected aseptically from the left median cubital vein using a 19-gauge needle and transferred to serum separator tubes labeled with unique identifiers. Samples were allowed to clot for 30 minutes at room temperature and centrifuged at 3500 RPM for 5 minutes.

### Laboratory procedure

Serum levels of gonadal hormones, including total testosterone, luteinizing hormone (LH), and follicle-stimulating hormone (FSH), were quantified using a chemiluminescent immunoassay on the Beckman Coulter DxI 800 AU automated analyzer (Beckman Coulter Inc., Danaher Corporation, Brea, CA, USA). The analytical performance of the system was validated through routine internal quality controls and external quality assurance (EQA) feedback from the Amhara Regional State Reference Laboratory.

Testosterone was measured using a competitive binding immunoenzymatic assay, in which light emission is inversely proportional to hormone concentration. LH and FSH were quantified via two-step sandwich immunoenzymatic assays, where light intensity is directly proportional to analyte concentration. All assays utilized multi-point calibration curves, and results were interpreted using the analyzer’s integrated luminometer.

### Data quality assurance and management

Comprehensive training was provided to data collectors and laboratory personnel covering the study objectives, ethical considerations (including informed consent, confidentiality, and participants’ rights), interview techniques, laboratory protocols, and quality assurance procedures. Under the supervision of the principal investigator, data collectors obtained detailed socio-demographic, clinical, and behavioral information, while trained laboratory technologists performed venous blood collection. The principal investigator oversaw the data collection process, ensuring data quality through routine supervision, real-time feedback, and verification of completeness and consistency. Cross-checking of specimen labels with participants’ unique identifiers was conducted to ensure traceability, and all data were systematically reviewed for accuracy and clarity. This manuscript was adhere to STROBE checklist and provide a completed STROBE checklist (S1 Data)

### Data analysis and interpretation

Data were entered, cleaned, coded, and analyzed using SPSS version 25 (SPSS Inc., Chicago, IL, USA). Normality of continuous variables was assessed using histograms and the Kolmogorov–Smirnov test; a p-value ≥0.05 was considered indicative of normal distribution. Descriptive statistics, including frequencies, percentages, means, and medians, were used to summarize the data, with results presented in tables and graphs.

Group comparisons for continuous variables were performed using independent t-tests. Binary logistic regression analysis was employed to identify independent predictors of hypogonadism, adjusting for potential confounders. Variables with a p-value ≤0.25 in bivariable analysis were included in the multivariable model. Prior to regression analysis, chi-squere test of the variables were checked and those variables that full filled the chi-square test assumptions were entered into the binary logistic regression. Also, multicollinearity was assessed using variance inflation factors (VIF), with all included variables showing VIF < 10.

### Ethical consideration

Ethical clearance was obtained from the ethical review committee of the School of Biomedical and Laboratory Sciences with the reference number SBMLS/762/2024. The study was conducted following the Declaration of Helsinki. A permission letter was obtained from UOGCSH. Participants were informed of the risks and benefits of the study; their right to withdraw anytime was maintained. Individuals’ abnormal laboratory test results on biochemical assessment and anthropometric measurement were linked to the responsible body for further diagnosis and treatment accordingly.

## Result

### Socio-demographic characteristics of the study participants

A total of 330 participants were enrolled in the study, yielding a response rate of 95.4%. Of these, 165 were individuals with clinically confirmed type 2 diabetes mellitus (T2DM), and 165 were age-matched, apparently healthy controls. The mean age of participants in the T2DM group was 57 ± 8.9 years, compared to 55 ± 8.6 years in the control group. Marital status analysis showed that the majority were married, comprising 92.7% of the T2DM group and 92.1% of controls. Most participants resided in urban areas, reported by 93.3% of those with T2DM and 98.2% of the control group (Table 1).

**Table 1:** Socio-demographic characteristics of study participants attending at UOG CSH, Gondar2024 (n = 330).

Variable	Categories	Case(n = 165)	Control(n = 165)	Total(n = 330)	p-value
		N (%)	N (%)	N (%)	
Age	35-44	19(11.5%)	24(14.5%)	43(13.0%)	0.186
	45-54	39(23.6%)	44(26.7%)	83(25.2%)	
	55-64	66(40.0%)	72(43.6%)	138(41.8%)	
	>65	41(24.8%)	25(15.2%)	66(20%)	
Marital status	Married	153(92.7%)	152(92.1%)	305(92.4%)	0.935
	Single	3(1.8%)	4(2.4%)	7(2.1%)	
	Divorced	6(3.6%)	7(4.2%)	13(3.9%)	
	Widowed	3(1.8%)	2(1.2%)	5(1.5%)	
Residence	Urban	154(93.3%)	162(98.2%)	316(95.8%)	0.052
	Rural	11(6.7%)	3(1.8%)	14(4.2%)	
Occupation	Merchant	33(20.0%)	40(24.2%)	73(22.1%)	0.000
	Farmer	17(10.3%)	3(1.8%)	20(6.1%)	
	Daily labor	7(4.2%)	4(2.4%)	11(3.3%)	
	Gov’t employee	36(21.8%)	80(48.5%)	116(35.2%)	
	Retired	58(35.2%)	33(20.0%)	91(27.6%)	
	Others	14(8.5%)	5(3.0%)	19(5.7%)	
Educational level	Unable to read and write	12(7.3%)	9(5.5%)	21(6.4%)	0.128
	Read and write	23(13.9%)	20(12.1%)	43(13.0%)	
	Primary school	58(35.2%)	41(24.8%)	99(30%)	
	Secondary school	38(23.0%)	55(33.3%)	93(28.2%)	
	Diploma and above	34(20.6%)	40(24.2%)	74(22.4%)	
Monthly income	<5000	109(66.1%)	102(61.8%)	211(63.9%)	0.422
	>5000	56(33.9%)	63(38.2%)	119(36.1%)	

Others* driver, NGO servant, private servant

### Behavioral characteristics of the study participants

Majority of, (62.4%) and (58.2%) T2DM patients and the control groups were alcohol drinker respectively. Most of, (90.3%) and (84.2%) of T2DM patients and the control group were high level stress respectively. (Table 2).

**Table 2:** Behavioral characteristics of the study participants at UOG CSH, Gondar 2024 (n = 330).

Variable	Categories	Case (n = 165)	Control(n = 165)	Total(n = 330)	P-value
		N (%)	N (%)	N (%)	
Alcohol consumption	Non-drinker	24(14.5%)	34(20.6%)	58(17.6%)	0.351
	Drinker	103(62.4%)	96(58.2%)	199(60.3%)	
	Ex-drinker	38(23.0%)	35(21.2%)	73(22.1%)	
Physical activity	Physically active	96(58.2%)	88(53.3%)	184(55.8%)	0.438
	Physically inactive	69(41.8%)	77(46.7%)	146(44.2%)	
Smoking	Smoker	16(9.7%)	26(15.8%)	42(12.7%)	0.136
	Non-smoker	149(90.3%)	139(84.2%)	288(87.3%)	
Stress	0-13(low)	48(29.1%)	37(22.4%)	85(25.8%)	0.382
	14-26(moderate)	102(61.8%)	112(67.9%)	214(64.8%)	
	27-40(high)	15(9.1%)	16(9.7%)	31(9.4%)	
Coffee drinking	Zero	40(24.2%)	33(20.0%)	73(22.1%)	0.606
	<2 cup per day	87(52.7%)	89(53.9%)	176(53.3%)	
	>2 cup per day	38(23.1%)	43(26.1%)	81(24.5%)	
Diet	<3 food item	13(7.9%)	33(20.0%)	46(13.9%)	0.000
	4-5 food item	60(36.4%)	93(56.4%)	153(46.4%)	
	>6 food item	92(55.7%)	39(23.6%)	131(39.7%)	

### Anthropometric and clinical characteristics of the study participants

The mean BMI level was 25.5 ± 3.21 and 25.2 ± 2.97 for T2DM and control groups respectively. Majority, (66.7%) and (68.5%) of T2DM and the control group were >94 cm WC respectively. Two-third (57.6%) of T2DM patients were known hypertensive patients whereas, 42.4% were not (Table 3).

**Table 3:** Anthropometric, and Clinical characteristics of the study participants at UOG CSH, Gondar 2024 (n = 330).

History of HTN	No	70(42.4%)		70(21.2%)	NA
	Yes	95(57.6%)		95(28.8%)	
SBP	<140mm/Hg	101(61.2%)	163(98.8%)	264(80%)	0.000
	≥140 mm/Hg	64(38.8%)	2(1.2%)	66(20%)	
DBP	<90mm/Hg	154(93.3%)	162(98.2%)	316(95.7%)	0.052
	≥90 mm/Hg	11(6.7%)	3(1.8%)	14(4.2%)	
BMI	Normal weight	77(46.7%)	84(50.9%)	161(48.8%)	0.625
	Over weight	72(43.6%)	69(41.8%)	141(42.7%)	
	Obese	16(9.7%)	12(7.3%)	28(8.5%)	
WC	<94 cm	55(33.3%)	52(31.5%)	107(32.4%)	0.073
	>94 cm	110(66.7%)	113(68.5%)	223(67.6%)	
Diabetic year	1-5year	44(26.7%)		44(13.3%)	NA
	6-10 year	67(40.6%)		67(20.3%)	
	11-15 year	30(18.2%)		30(9.1%)	
	>16 year	24(14.5%)		24(7.3%)	
Diabetic complication	Nephropathy	2(1.2%)		2(0.6%)	NA
	Retinopathy	10(6.1%)		10(3.0%)	
	Neuropathy	4(2.4%)		4(1.2%)	
	No complication	149(90.3%)		149(45.1%)	
Type of medication	Metformin	51(30.9%)		51(15.5%)	NA
	Metformin and Glibinclamide	52(31.5%)		52(15.6%)	
	Insulin	40(24.2%)		40(12.1%)	
	Metformin and insulin	18(10.9%)		18(5.5%)	
	Glibinclamide	4(2.4%)		4(1.2%)	

*Statistically significant NA = not applicable HTN = hypertension SBP = systolic blood pressure DBP = diastolic blood pressure BMI = body mass index WC = waist circumference

### Biochemical profile of the study participants

The mean FBS level for T2DM and control group was 152.6 ± 57.2 and 106.6 ± 14.2 respectively. (Table 4).

**Table 4:** Biochemical profile of study participants attending at UOG CSH, Gondar 2024 (n = 330).

Variable	Categories	Case(n = 165)	Control(n = 165)	Total(n = 330)	Mean of test value for T2DM&control	Median of test value for T2DM&control	p-value
		N (%)	N (%)	N (%)			
Total testosterone(ng/ml)	Low	50(30.3%)	12(7.3%)	62(18.8%)	2.68 ± 1.52& 3.50 ± 1.48		0.00
	Normal	113(68.5%)	151(91.5%)	264(80%)			
	High	2(1.2%)	2(1.2%)	4(1.2%)			
FSH(IU/L)	Normal	136(82.4%)	148(89.7%)	284(86.1%)	11.9 ± 10.5& 10.6 ± 7.8		0.080
	High	29(17.6%)	17(10.3%)	46(13.9%)			
LH(IU/L)	Normal	118(71.5%)	114(69.1%)	232(70.3%)	7.65 ± 10.5&7.7 ± 5.1		
	High	47(28.5%)	51(30.9%)	98(29.7%)			
FBS(mg/dl)	Hypoglycemic	6(3.6%)	-----	6(1.8%)	152.56 ± 57.2 & 106.6 ± 14.21		NA
	Normoglycemic	52(31.5%)	165(100%)	217(65.8%)			
	Hyperglycemic	107(64.8%)	-----	107(32.4%)			

### Lipid profile of the study participants

The mean level TC for T2DM patients and the control groups were 133.96 ± 42.5&143.9 ± 42.13 respectively. Most of T2DM and the control groups were normal level of TC, TG, and LDL-c (Table 5).

**Table 5:** Lipid profile of the study participants attending at UOG CSH, Gondar 2024 (n = 330).

TC(mg/dl)	Normal	155(93.9%)	148(89.7%)	303(91.8%)	133.96 ± 42.5&143.9 ± 42.13		0.468
	High	10(6.1%)	17(10.3%)	32(8.2%)			
TG(mg/dl)	Normal	113(68.5%)	118(71.5%)	231(70%)		121 ± 78 & 119 ± 71	0.147
	High	52(31.5%)	47(28.5%)	99(30%)			
HDL_c(mg/dl)	low	112(67.9%)	128(77.6%)	240(72.7%)		31 ± 1 & 33 ± 12	0.083
	Normal	53(32.1%)	37(22.4%)	90(27.3%)			
LDL_c(mg/dl)	Normal	153(92.7%)	148(89.7%)	301(91.2%)	82.96 ± 29.47&89.7 ± 31.8		0.960
	High	12(7.3%)	17(10.3%)	29(8.8%)			

Note:*Statistically significant FSH = follicle stimulating hormone LH = luteinizing hormone FBS = fasting blood sugar TC = total cholesterol TG = triglyceride HDL-c = high density lipoprotein cholesterol LDL = low density lipoprotein

### Prevalence of hypogonadism

**Fig 1:**
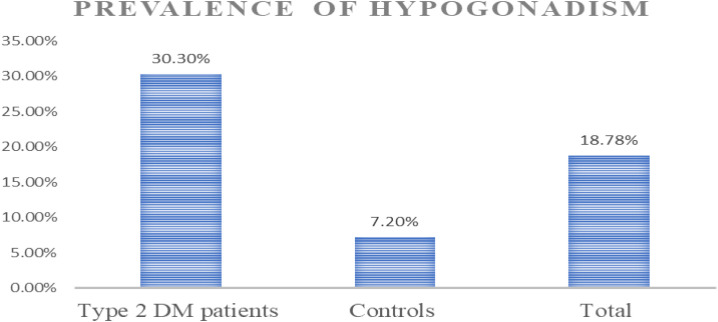
Prevalence of Hypogonadism among the study participants, T2DM patients and apparently healthy control group from UOG CSH Gondar 2024.

The overall prevalence of hypogonadism among the study population was 18.8% (95% CI: 14.8–23.0). Stratified analysis revealed a significantly higher prevalence in the type 2 diabetes mellitus (T2DM) group at 30.3% (95% CI: 23.4–37.9) compared to 7.2% (95% CI: 3.8–12.4) in the apparently healthy control group. (Fig 1).

The positive response rate of the study participants for ADAM questionnaire was 149(90.3%) and 55(33.3%) for T2DM and control group respectively (Table 6).

**Table 6:** Response rate of ADAM questionnaire for the study participants (n = 330).

Status	Case	Control	Total
	N	N	
ADAM positive	149(90.3%)	55(33.3%)	204(61.8%)
ADAM negative	16(9.7%)	110(66.7%)	126(38.2%)
Total	165(100%)	165(100%)	330(100%)

### Mean comparison of selected sex hormones among T2DM and control groups

There was a statistically significant difference in mean total testosterone levels between patients with T2DM and the healthy control group (p-value = 0.000) (Table 7).

**Table 7:** Independent T test for the comparison of selected sex hormone mean difference between T2DM patients and control groups in UOG CSH, Gondar 2024(n = 330).

Variable	T2DM	Control	Mean difference	p-value
	X ± SD	X ± SD		
Total testosterone(ng/ml)	2.68 ± 1.52	3.50 ± 1.48	−0.81673	0.000^*^
FSH(IU/L)	11.9 ± 10.5	10.6 ± 7.8	1.30	0.204
LH(IU/L)	7.65 ± 5.28	7.7 ± 5.07	−0.129	0.822

* Statistically significant X = mean SD = Standard deviation FSH = follicle stimulating hormone LH = luteinizing hormone

### Factors associated with hypogonadism among the study participants

Variables who full fill chi-square test assumption and with a p-value < 0.25 in the bivariable logistic regression analysis were subsequently entered into the multivariable logistic regression model. Prior to multivariable analysis, multicollinearity among the independent variables was assessed using variance inflation factor (VIF) diagnostics, with all variables demonstrating VIF values below the threshold of 10, indicating absence of significant multicollinearity. Multivariable logistic regression identified age, HDL-c, presence of DM, and BMI as independent factors significantly associated with hypogonadism among the overall study population (Table 8).

**Table 8:** Bivariable and multivariable binary logistic regression analysis result for factors associated with hypogonadism among study participants (n = 330).

Variable	Category	Hypogonadism		COR(95%CI)	AOR (95% CI)	P-value
		Yes	No			
Age category	35-44	4(9.3%)	39(90.7%)	1	1	
	45-54	10(12%)	73(88%)	1.33(0.383-4.426)	1.96(0.476-8.050)	0.352
	55-64	23(16.7%)	115(83.3%)	1.95(0.618-5.843)	3.39(0.836-13.763)	0.087
	>65	25(37.9%)	41(62.1%)	5.94(1.845-18.186)	10.3(2.417-44.364)	0.001^*^
BMI	Normal weight	20(12.9%)	138(87.1%)	1	1	
	Overweight	32(22.4%)	111(77.6%)	2.0(1.122-3.817)	1.94(0.841-4.453	0.120
	Obese	10(34.5%)	19(65.5%)	3.63(1.59-9.67)	4.25(1.201-15.006)	0.025^*^
Alcohol	Non-Drinker	9(15.5%)	49(84.5%)	1	1	
	Drinker	32(16.1%)	167(83.9%)	1.04(0.466-2.334)	1.10(0.406-2.996)	0.847
	Ex-drinker	21(28.8%)	52(71.2%)	2.19(0.918-5.264)	3.06(0.986-9.468)	0.053
Stress	Low	18(21.2%))	67(78.8%)	1	1	
	Moderate	41(19.2%)	173(80.8%)	0.88(0.474-1.643)	1.43(0.641-3.179)	0.384
	High	3(9.7%)	28(90.3%)	0.39(0.109-1.462)	0.34(0.070-1.638)	0.178
Smoking	Smoker	3(7.1%)	39(92.9%)	0.29(0.089-1.00)	0.27(0.063-1.124)	0.072
	Non-smoker	59(20.5%)	229(79.5%)	1	1	
History of HTN	No	33(14.0%)	202(86%)	1	1	
	Yes	29(30.5%)	66(69.5%)	2.69(1.519-4.761)	0.40(0.153-1.063)	0.066
Presence of DM	No	12(7.3%)	153(92.7%)	1	1	
	Yes	50(30.3%)	115(69.7%)	5.54(2.823-10.886)	7.01(2.628-18.723)	0.000^*^
SBP	<140mmHg	43(16.3%)	221(83.7%)	1	1	
	>140mmHg	19(28.8%)	47(71.2%)	2.08(1.112-3.882)	1.03(0.410-2.566)	0.957
DBP	<90mmHg	57(18.0%)	259(82.0%)	1	1	
	>90mmHg	5(35.7%)	9(64.3%)	2.52(0.815-7.816)	1.40(0.333-5.925)	0.643
Continues independent variable						
Variable			COR(95% CI)		AOR(95% CI)	P-value
Waist circumference			1.02(0.988-1.052)		0.97(0.930-1.016)	0.212
Fasting blood sugar			1.01(1.004-1.014)		1.01(1.000-1.014)	0.059
Follicle stimulating hormone			1.02(0.996-1.049)		1.01(0.975-1.044)	0.600
Total cholesterol			1.00(0.989-1.002)		1.01(0.997-1.027)	0.121
High density lipoprotein cholesterol			0.95(0.923-0.986)		0.95(0.904-0.998)	0.042^*^
Low density lipoprotein cholesterol			0.99(0.981-1.000)		0.99(0.971-1.013)	0.430

* Statistically significant HTN= Hypertension COR=crude odds ratio

CI=confidence interval AOR=adjusted odds ratio

### Factors associated with hypogonadism among T2DM patients

The independent variable like Age, physical activity, WC, FBS, HDL_c, LDL_c, and BMI with p-value less than 0.25 in bivariable logistic regression model were taken into multivariable logistic regression model. After multivariable analysis FBS, Age and BMI showed a significant association with hypogonadism among T2DM (Table 9).

**Table 9:** Bivariable and multivariable binary logistic regression analysis result for factors associated with hypogonadism among diabetes mellitus patients (n = 165).

Variable	Category	Hypogonadism		COR(95%CI)	AOR(95%CI)	P-Value
		Yes	No			
Physical activity	Physically active	25(26%)	71(74%)	1	1	0.344
	Physically inactive	25(36.2%)	44(63.8%)	1.61(0.826-3.153)	1.44(0.674-3.093)	
Age category	35-44	4(21.1%)	15(78.9%)	1	1	
	45-54	7(17.9%)	32(82.1%)	0.82(0.208-3.238)	1.08(0.228-5.067)	*0.926*
	55-64	19(28.8%)	47(71.2%)	1.52(0.445-5.160)	2.27(0.539-9.536)	*0.264*
	>65	20(48.8%)	21(51.2%)	3.57(1.011-12.610)	6.03(1.299-27.985)	*0.022* ^*^
BMI category	Normal weight	16(20.8%)	61(79.2%)	1	1	
	Overweight	25(34.7%)	47(65.3%)	2.03(0.974-4.224)	1.82(0.748-4.439)	0.187
	Obese	9(56.3%)	7(43.7%)	4.90(1.582-15.186)	5.46(1.287-23.141)	0.021^*^
Continuous variables						
Category	COR(95%CI)			AOR(95%CI)		P-value
WC	1.03(0.991-1.069)			0.98(0.939-1.032)		0.517
FBS	1.01(1.003-1.021)			1.02(1.007-1.028)		0.001^*^
HDL-c	0.96(0.926-0.999)			0.98(0.939-1.033)		0.535
LDL-c	0.99(0.978-1.002)			0.99(0.981-1.012)		0.656

* Significant association 1=reference

Abbreviations WC = waist circumference DBP = diastolic blood pressure FBS = fasting blood sugar HDL-c = high density lipoprotein LDL-c = low density lipoprotein BMI = body mass index COR = crude odds ratio AOR = adjusted odds ratio CI = confidence interval.

## Discussion

This study aimed to assess the prevalence and associated factors of hypogonadism among T2DM patients compared with age matched apparently healthy control groups. Based on the operational definition of hypogonadism, the prevalence of hypogonadism among the study participants were 18.78[95% CI: 14.8%, 23.0%], whereas, the prevalence of hypogonadism among T2DM patients and apparently healthy was 30.3% [95% CI: 23.4%, 37.9%] and 7.3% [95% CI: 3.8%, 12.4%] respectively. In multivariable analysis factors such as Age, BMI, FBS, having DM, and HDL were significantly associated with hypogonadism.

The prevalence of hypogonadism among T2DM in this study was 30.3%[95% CI: 23.4%, 37.9%] which was consistent with the cross-sectional study conducted in Korea(34.9%) [22], Pakistan(24.8%) [23], western India (26%) [23], Saudi Arabia(36.5%) [24], Nigeria(30%) [25], Ethiopia(23.5%) [26], and a case control study conducted in Accra Ghana(35.2% for case and 6.7% for control) [27]. However, this finding was higher than the cross sectional study conducted in Spain(17.2%) [28], Iran(11.8%) [15], comparative cross sectional study conducted in western India(15%) and India(17.3%) [29,30]. On the other hand, this finding was lower than the cross sectional study conducted in Australia (43%) [31], India(42%) [32], Egypt(40%) [33], comparative cross sectional study conducted in Sudan(78% for T2DM and 10% for control groups) [16], and retrospective study conducted in Mali(65.45%) [34].

Variations in hypogonadism prevalence across studies may be attributed to differences in diagnostic criteria, population characteristics(genetic factor, cultural difference, and life style factor), and methodologies [35]. Studies in Mali (<2.2 ng/mL) and Sudan (<2.5 ng/mL) used higher testosterone cutoffs than our study (<1.75 ng/mL), potentially leading to prevalence discrepancies.[34,36]. Since, the present study includes respondents with total testosterone below 1.75ng/ml, values higher than this number where considered as eugonadal that lowers the number of cases who have total testosterone value between 1.75ng/ml-2.5ng/ml. Measurement differences, such as total versus free testosterone, also contribute, as seen in an Indian study using free testosterone. Population factors, including higher mean age in Australia (65 ± 1 years) and Sudan (61.6 ± 9.5 years) [16,31], and higher BMI in Egypt (28.18 ± 2.94 vs. 25.5 ± 3.21 in our study), may influence findings. Methodological differences, such as smaller sample sizes in Iran (n = 96) and Western India (n = 100), reduce statistical power [15,29].

The mean level of total testosterone in T2DM patients were significantly lower than the control group. The plausible justification for this difference might be due to the presence of insulin resistance in T2DM, this condition could impaired the normal Leyding cell function and cause low testosterone for T2DM patients [27]. The significantly lower mean level of total testosterone in T2DM patient as compared the control group in this study (2.68 ± 1.52 vs. 3.504 ± 1.48 ng/ml) was consistent with the study done in Nigeria, India, Egypt, and Sudan [30,36–38].

There was no statistically significant mean FSH difference between T2DM and control groups. This finding was consistent with the study done in India [29] and Ghana [27]. However, this finding was contraindicated with the finding of India [30] and Sudan [16], the possible reason for this variation might be difference in genetic factors, life style factors, methodological difference, and population difference.

There was no statistically significant mean LH difference between T2DM and the control group. This finding was consistent with the study done in Maharashtra India [29], India [30]. However, this finding was contra indicated with the finding of Ghana and Sudan [16,27], the possible reason for this variation might difference in genetic factors, life style factors.

It was found that obese individuals of the Study participants and T2DM patients were 4.25 and 5.46 more likely to have hypogonadism than normal weight (AOR = 4.25, 95% CI: 1.201–15.006) and (AOR = 5.46, 95% CI: 1.287–23.141) respectively. Similar finding were found in the study conducted in Nigeria and Egypt [25,33]. The possible reason might be, sedentary life style of Ethiopian population leads to obesity and, in obese individual there is an excess formation of aromatase enzyme, leptin hormone and cytokines (TNFα and IL-1β). So, the aromatase enzyme convert serum testosterone levels to estradiol this conversion leads to lower blood testosterone levels and acts as negative feedback on the hypothalamic-pituitary-gonadal axis (HPG) and gonadotropin-releasing hormone [7,8]. Even though, excess leptin hormones are produced in adipose tissue, obesity impaired its action on the HPG axis and cause reduced LH secretion and testosterone release [39]. Also, adipo-cytokines (TNF-α and IL-1β) reduce hypothalamic GnRH, LH, and testosterone secretion [11].

Presence of DM and FBS was also significantly associated with hypogonadism among the study participants. Being diabetic patient have 7.01 odds of having hypogonadism than non-diabetic (AOR = 7.01, 95% CI: 2.628–18.723). This could be due to the effect of hyperglycemia on the microvasculature of the testis by attenuated the function of Leyding cells to produce testosterone. Additionally, if glucose is not reaching the cells because of insulin insensitivity, there may not be enough energy generated for the various metabolic processes involved in maintaining testosterone levels [40,41].

Age category >65 year individuals had 10.3 and 6.03 odds of having hypogonadism than the age group of 35–44(AOR = 10.3, 95% CI: 2.417–44.364) and (AOR = 6.03, 95% CI: 1.299–27.985) respectively. The finding of this result was supported by the studies conducted in Spain [28], India [42], Saudi Arabia [24], Egypt [33], and Sudan [36]. This is due to aging reduces outflow of GnRH and the efficacy of endogenous LH-mediated testosterone secretion [43,44].

It was found that a unit increase in FBS level increases the risk of hypogonadism by 1.02(AOR = 1.02, 95% CI: 1.007, 1.028). This finding was supported by the studies conducted in Spain, Korea, Ghana, and Ethiopia [22,26–28]. This could be due to poorly controlled diabetes of Ethiopian population, the effect of hyperglycemia on the microvasculature of the testis by attenuated the function of Leyding cells to produce testosterone. Additionally, if glucose is not reaching the cells because of insulin insensitivity, there may not be enough energy generated for the various metabolic processes involved in maintaining testosterone levels [40,41].

It was found that a unit increase in HDL-c level decreasing the risk occurrence of hypogonadism by (AOR = 0.95. 95% CI: 0.898–0.993). The finding of this study was supported by the study conducted in Sudan [36]. This might be linked to improved insulin sensitivity when the level of HDL-c increase. So, the better insulin regulation can positively impact testosterone production and decrease the risk of metabolic disorder that can lead to hypogonadism [45].

## Conclusion

In this study we conclude that the prevalence of hypogonadism among T2DM was higher than the control group. In addition to this the mean level of total testosterone was lower in T2DM patients than the control whereas, the mean level of FSH and LH was not statistically different between T2DM and apparently healthy control groups. Study variables like BMI, FBS, Age, presence of DM and HDL-c were significantly associated with hypogonadism among the study participants and T2DM patients.

### Recommendation

Health policy and program implementers should prioritize the development and implementation of structured weight loss programs for obese individuals with T2DM, as this study identifies obesity as a major contributing factor to hypogonadism. T2DM patients are encouraged to engage in regular physical activity to mitigate central obesity and achieve sustainable weight reduction. Furthermore, given the limited research on this topic in Ethiopia, future studies with larger sample sizes and longitudinal follow-up are recommended to further elucidate the underlying factors associated with hypogonadism in this population.

### Strength and limitation of the study

This study is the first in the region to incorporate both the Androgen Deficiency in Aging Males (ADAM) questionnaire and laboratory assessments for a comprehensive evaluation of hypogonadism. However, several limitations should be acknowledged. The cross-sectional design precludes causal inferences regarding the relationship between hypogonadism and T2DM. Even though, this study measures morning level of testosterone due to reagent constraint, hypogonadism prevalence was determined using a single testosterone measurement, which may not fully capture hormonal fluctuations. Similarly, a single FBS measurement was used to assess glycemic status in the control group, potentially limiting accuracy. The absence of free testosterone measurement in the study setting, considered the gold standard for diagnosing hypogonadism, represents another constraint. Lastly, social desirability bias may have influenced self-reported responses due to the sensitivity of the study topic.

## Supporting information

S1 Data This is supplementary legend that contain STROBE checklist. (DOC)

S2 Data Questionnaire. (DOCX)

S3 Data Raw Data of our Manuscipt. (XLSX)

## Abbreviations

AIDS: Acquired Immunodeficiency Syndrome
AOR: Adjusted odds ratio
BMI: Body Mass Index
CI: Confidence interval
COR: Crude odds ratio
DM: Diabetes Mellitus
ED: Erectile Dysfunction
FBS: Fasting blood sugar
FSH: Follicle stimulating hormone
GnRH: Gonadotropin Releasing Hormone
HPG-a: Hypothalamic Pituitary Gonadal Axis
HDL-c: High Density Lipoprotein
HIV: Human Immunodeficiency Virus
HTN: Hypertension
IDF: International Diabetic Federation
LDL-c: Low Density Lipoprotein
LH: Luteinizing Hormone
SHBG: Sex Hormone Binding Globulin
TRT: Testosterone Replacement Therapy
TG: Triglyceride
TC: Total Cholesterol
T2DM: Type 2 Diabetes Mellitus
WC: Waist Circumference
hcG: Human chorionic gonadotropin
