# Supplementary material for: Hypogonadism and its associated factors among adult male type 2 diabetes mellitus patients at the university of gondar comprehensive specialized hospital, Northwest Ethiopia 2024: A comparative cross-sectional study
Source: PLoS One. 2025 Aug 14;20(8):e0329784. doi: 10.1371/journal.pone.0329784 (PMC12352672; doi:10.1371/journal.pone.0329784)
Supplement: S2 Data — (DOCX) [file pone.0329784.s002.docx]

**Questionnaire**

Part I- Questionnaire related with socio-demographic characteristics

Please write your answer in the space provided in the column of participant response.

| Question no_ | Questions | Participant response | |
| --- | --- | --- | --- |
| 1. | How old are you? |  | Year |
|  |  |  |  |
| 2. | What is your marital status? | 1. Married 2. Single 3. Divorced 4. Widowed | |
| 3. | What is your educational level? | 1. no formal education 2. Elementary and less 3. secondary school 4. diploma 5. degree and above | |
| 4. | What is your average monthly income per ETB? | 1. 5000 and below 2. 5000 and above | |

Part II: Questionnaire related with behavioral characteristics

| 5. | Do you perform physical exercise? If yes, please specify the frequency and duration | 1. day per week 2. minute per day 3. No routine exercise | | | | |  |
| --- | --- | --- | --- | --- | --- | --- | --- |
| 6. | Do you consume alcohol(beer, wine, sprits, tella, areki, tej? if yes please specify the  frequency and Duration | 1. per day 2. day per week 3. Never drink 4. I have stopped drinking alcohol | | | | |  |
| 7. | Do you smoke cigarette? if yes please specify the frequency and the amount | 1. days per week 2. per day 3. Never smoked 4. I have stopped smoking | | | | |  |
| 8. | Do you have a habit of drinking coffee? | 1. No 2. <2cups per day 3. >2cups per day | | | | |  |
| 9. | Foods that you ate yesterday from morning to night | 1. Morning 2. Lunch 3. Dinner | | | | |  |
| 10. | Questions related with perceived stress scale? | Scale | | | | |  |
|  |  | 0 | 1 | 2 | 3 | 4 | |
| 10.1 | In the last month, how often have you been upset because of something that  happened unexpectedly? |  |  |  |  |  | |
| 10.2 | In the last month, how often have you felt that you were unable to control the important  things in your Life? |  |  |  |  |  | |
| 10.3. | In the last month, how often  have you felt nervous and stressed‖? |  |  |  |  |  | |
| 10.4. | In the last month, how often have you felt confident about your ability to handle y o u r  p e r s o n a l problems? |  |  |  |  |  | |
| 10.5. | In the last month, how often have you felt that things were  going your way? |  |  |  |  |  | |
| 10.6. | In the last month, how often have you found that you could not cope with all the things  that you had to do? |  |  |  |  |  | |
| 10.7 | In the last month, how often have you been able to control  irritations in your life? |  |  |  |  |  | |
| 10.8 | In the last month, how often  have you felt that you were on top of things? |  |  |  |  |  | |
| 10.9 | In the last month, how often have you been angered because of things that were outside of  your control? |  |  |  |  |  | |
| 10.10 | In the last month, how often have you felt difficulties were piling up so high t h a t y o u  c o u l d n o t Overcome them? |  |  |  |  |  | |

Part III Androgen Deficiency in the Aging Male (ADAM) questionnaire about symptoms of low testosterone and encircle your answer.

| No_ | Questions | Answers |
| --- | --- | --- |
| 1 | Has there been deterioration in your work performance? | Yes  No |
| 2 | Do you have a lack of energy? | Yes  No |
| 3 | Do you have a decrease in strength and/or endurance? | Yes  No |
| 4 | Have you noticed a recent deterioration in your ability to  Play sports? | Yes  No |
| 5 | Have you noticed a decreased "enjoyment of life" | Yes No |
| 6 | Are you sad and/or grumpy? | Yes No |
| 7 | Are you falling asleep after dinner? | Yes No |
| 8 | Have you lost height? | Yes  No |
| 9 | Do you have a decrease in libido (sex drive)? | Yes  No |
| 10 | Are your erections less strong? | Yes  No |

Part IV: checklist to collect medical record for case group

| No_ | Type of data from medical record | Replay from the record | Remark |
| --- | --- | --- | --- |
|  | Type of treatment | Metformin  Metformin with glibinclamide Insulin  Metformin with insulin  Glibinclamide  Others________ |  |
|  | Diabetic year | _______-month/year |  |
|  | Diabetic nephropathy | Yes  No |  |
|  | Diabetic Retinopathy | Yes  No |  |
|  | Diabetic neuropathy | Yes  No |  |
|  | Other |  |  |
